# Ursodeoxycholic acid production by *Gibberella zeae* mutants

**DOI:** 10.1186/s13568-022-01446-2

**Published:** 2022-08-08

**Authors:** Vyacheslav Kollerov, Marina Donova

**Affiliations:** Federal Research Center Pushchino Center for Biological Research of the Russian Academy of Sciences, G.K. Skryabin Institute of Biochemistry and Physiology of Microorganisms, Russian Academy of Sciences, Prospekt Nauki, 5, 142290 Pushchino, Moscow Region Russia

**Keywords:** *Gibberella zeae*, Protoplasts, Mutagenesis, 7β-hydroxylation, Lithocholic acid, Ursodeoxycholic acid

## Abstract

Ursodeoxycholic acid (UDCA) is a highly demanded pharmaceutical steroid widely used in medicine. An ascomycete *Gibberella zeae* VKM F-2600 is capable of producing UDCA by 7β-hydroxylation of lithocholic acid (LCA). The present study is aimed at the improvement of the fungus productivity. The original procedures for the protoplast obtaining followed by UV mutagenesis and screening of ketoconazole-resistant mutant clones have been applied. The highest yield of *G. zeae* protoplasts was obtained when using the mycelium in the active growth phase, ammonium chloride as an osmotic stabilizer and treatment of the fungal cells by the lytic enzymes cocktail from *Trichoderma hurzanium*. The conditions for effective protoplast regeneration and the UV-mutagenesis were found to provide 6–12% survival rate of the protoplasts with superior number of possible mutations. Three of 27 ketoconazole-resistant mutant clones obtained have been selected due to their increased biocatalytic activity towards LCA. The mutant *G. zeae* M23 produced 26% more UDCA even at relatively high LCA concentration (4 g/L) as compared with parent fungal strain, and the conversion reached 88% (w/w). The yield of UDCA reached in this study prefers those ever reported. The results contribute to the knowledge on ascomycete mutagenesis, and are of importance for biotechnological production of value added cholic acids.

**Graphical Abstract:**

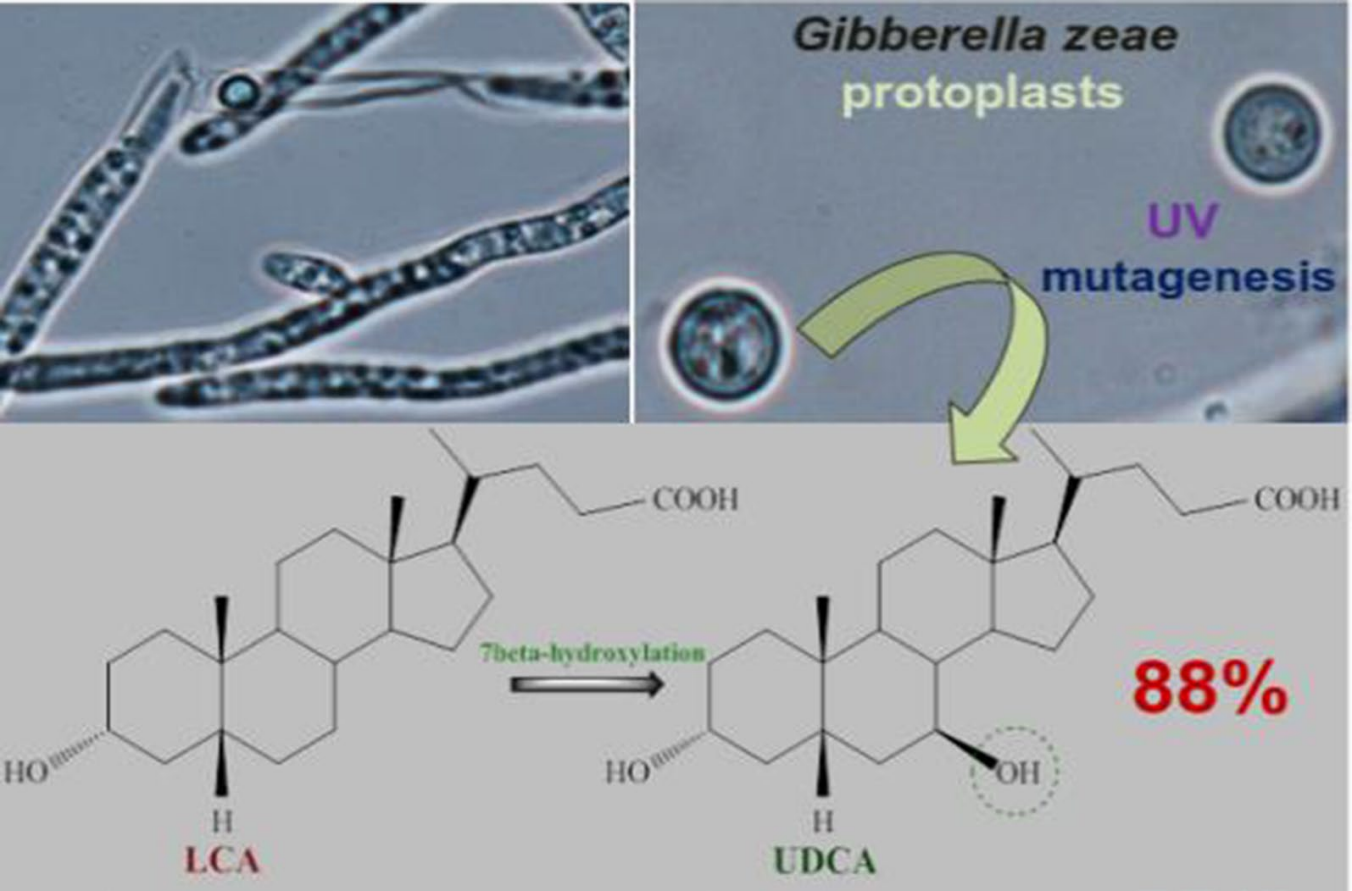

## Key points

- Efficient procedures for production and regeneration of Gibberella zeae protoplasts were determined.
- Fungal mutants were obtained with elevated 7β-hydroxylase activity.
- Mutant G. zeae M23 almost fully converts LCA (4 g/L) to UDCA.

## Introduction

Bile acids (BAs) are 3α-hydroxy 5β-steroids of 24 carbons. The number, position and stereochemistry of the hydroxyl groups in their steroid core determine their solubility, amphipathic properties, biochemical features and biological activity. In mammals, bile acids are synthesized from cholesterol and play an important role in digestion (Hofmann and Hagey 2008).

Ursodeoxycholic acid (3α,7β-dihydroxy-5β-cholanic acid, UDCA) belongs to the so-called secondary bile acids, and is formed from the primary acids such as cholic acid (CA) or chenodeoxycholic acid (CDCA) by intestinal bacteria (Prabha and Ohri 2006; Begley et al. 2005). UDCA (also known as ursodiol) occupies a special place among the bile acids due to its superior therapeutic properties. This active ingredient of the bear (“urso”) bile has been used in traditional Chinese medicine for more than 3000 years and was shown to possess anti-inflammatory, antioxidant, immunomodulatory and anti-apoptotic properties (Abdulrab et al. 2020). Nowadays, UDCA is widely used in medicine for dissolving gallstones, treatment and prevention of cholestasis, sclerosing cholangitis, hepatitis and liver cirrhosis and other diseases in gastroenterology and hepatology (Bortolini et al. 1997; Angulo et al. 1999; Ikegami and Matsuzaki 2008; Philipp 2011). It also exhibits protective effects against the colon cancer (Guzior and Quinn 2021). There is evidence of a beneficial effect of UDCA in the treatment of respiratory diseases (Işık et al. 2017).

Currently, industrial production of UDCA is based on a multi-stage, ecologically non-friendly chemical synthesis from CA isolated from cattle bile (Sawada et al. 1982). The synthesis requires several steps of protection and deprotection of the hydroxyl groups, and the overall UDCA yield does not exceed 30% (Tonin and Arends 2018).

**Fig. 1:**
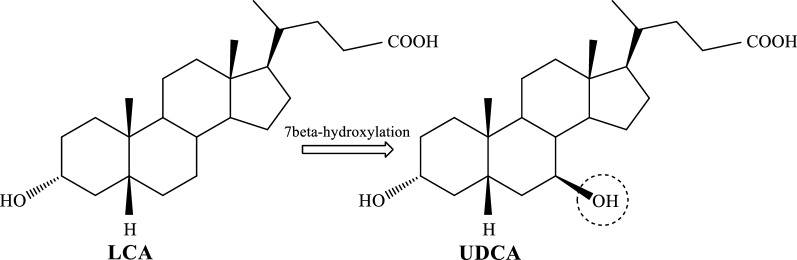
Conversion of LCA to UDCA

An alternative approach includes microbial transformation or chemoenzymatic synthesis from CA, or CDCA (Tonin and Arends 2018; Wang et al. 2016). Using lithocholic acid (LCA) as a starting material for bioconversion (Fig. 1) provides a single-step UDCA production (Moriarty et al. 2014).

Filamentous fungi are well-known biocatalysts of regio- and stereospecific hydroxylation of androstane and pregnane steroids (Nassiri-Koopaei and Faramarzi 2015; Durairaj et al. 2016; Girvan and Munro 2016; Kristan and Riˇzner 2012), however their biocatalytic potential with respect to BAs is less studied. The strains of *Rhizoctonia solani*, *Helicostilum piriforme*, *Cunninghamella blakesleeana* and *Curvularia lunata* have been reported to oxyfunctionalize LCA mainly at positions C-12 and C-15 (Carlstroem et al. 1981; Hayakawa et al. 1980; Kulprecha et al. 1985). The 7β-hydroxylase activity towards LCA was described for *Fusarium equizeti* M-41 but the yield of UDCA was low even under optimized conditions (Sawada et al. 1982).

Previously, we have conducted a wide screening of filamentous fungi for their activity towards LCA and selected the strains of the genera *Bipolaris, Gibberella, Cunninghamella, Cochliobolus* and *Fusarium* with 7β-hydroxylase activity. Among them, the ascomycete *Gibberella zeae* VKM F-2600 (anamorph *Fusarium graminearum*) was chosen because of its superior potential, allowing up to 30% UDCA to be obtained from LCA (1 g/L) even under non-optimized conditions. Further optimization of the bioconversion conditions provided a threefold increase in the UDCA yield, but a lower bioconversion rate was observed at the elevated substrate concentrations (Kollerov et al. 2013).

Bioconversion enhancement is possible by fungal strain improvement that can be achieved by mutagenesis (Solis et al. 1996). The resistance to azole fungicide ketoconazole (inhibitor of cytochrome P 450 enzymes) is often used as a selective marker (Lu et al. 2007). A mutation in the gene encoding hydroxylase (CYP) enzyme synthesis can lead to its overexpression providing the resistance of fungal cells to ketoconazole (Lu et al. 2007; Wilmańska et al. 1992).

The main obstacle to successful mutagenesis of filamentous fungi is often low efficiency of the action of the mutagenic factors on fungal cells because of the complex structure of the cell wall and the multinuclear nature of filamentous fragments (Garcia-Rubio 2020). The use of protoplasts,—single mononuclear cells devoid of a cell wall and surrounded only by a cytoplasmic membrane, can facilitate genetic manipulation and mutagenesis of filamentous fungi and often plays a significant role in strain improvement (Roth and Chilvers 2019; Zhou et al. 2008). In turn, effective protoplast production, release and mutagenesis depend on many factors including fungal species, mycelium state and protoplasting conditions (osmotic stabilizer, temperature, pH, digestion time, lytic enzyme concentration and others) whether a protoplast is mononucleate, binucleate or multinucleate (Kumari and Panda, 1992; Coelho et al. 2010; Ren et al. 2018).

This study aimed to improve *G. zeae* VKM F-2600 strain for enhancement of LCA to UDCA bioconversion. The procedures for effective protoplast isolation and regeneration were developed and the mutants with increased 7β-hydroxylase activity were obtained.

## Materials and methods

### Chemicals

Lithocholic acid (3α-hydroxy-5β-cholanic acid, LCA) and ursodeoxycholic acid (3α,7β-dihydroxy-5β-cholanic acid, UDCA) were obtained from ACROS Organics (New Jersey, USA). Yeast extract was purchased from Difco (Franklin Lakes, USA), lysing enzymes from *Trichoderma harzianum* and corn steep solids—from Sigma-Aldrich (St. Louis, USA). All other reagents were of the best quality grade from commercial suppliers.

### Microorganism and cultivation

*G. zeae* VKM F-2600 was obtained from the All-Russian Collection of Microorganisms at the Institute of Biochemistry and Physiology of Microorganisms, Russian Academy of Sciences (VKM IBPM RAS).

The fungus was routinely maintained on wort-agar slants. To obtain the first generation mycelium, the spore suspension from one agar slant (4 weeks old) was inoculated in 50 mL of the medium A containing (g/L): potato starch—45, corn steep solids—10, yeast extract—3, K_2_HPO_4_—2, KH_2_PO_4_—16, (pH 6.5) aerobically on a rotary shaker (220 rpm) at 28 °C for 24 h in Erlenmeyer flasks (750 mL).

### Protoplast obtaining

The *G. zeae* mycelium grown in medium No 1–6 (Table 1) was separated from the culture liquid by filtration through gauze, washed with distilled water and centrifuged at 3000×*g* for 10 min. 100 mg of wet mycelium was resuspended in 1 mL of 0.1 M potassium-phosphate buffer (pH 6.0) supplemented with 2.5–15 mg of lytic enzymes complex from *T. harzianum* and 0.6–1.2 M osmostabilizer (KCl, NH_4_Cl, MgSO_4_ or sucrose). Incubation was carried out on a rotary shaker (220 rpm) at 28 °C for 1–15 h. The presence of protoplasts was controlled by H550s optical microscope (Nikon, Melville, USA). Protoplasts were separated from hyphal debris by filtration through the glass filter followed by centrifugation at 45,000×*g* for 15 min. The obtained sediment was re-suspended in 0.8 mL of 0.1 M potassium-phosphate buffer with an osmostabilizer.

**Table 1:** Influence of medium composition on *G. zeae* protoplast formation

Medium no	Nutrient components, g/L							Protoplast yield/mL
	Sucrose	Glucose	Yeast extract	Soy flour	Peptone	KH_2_PO_4_	MgSO_4_	
1	20	–	–	–	–	1	0,5	0.3 (± 0.02) × 10^4^
2	20	–	5	–	–	1	0,5	0.2 (± 0.01) × 10^5^
3	–	20	5	–	–	1	0,5	0.6 (± 0.03) × 10^5^
4	–	20	–	10	–	1	0,5	0.7 (± 0.04) × 10^3^
5	–	20	–	–	10	1	0,5	0.2 (± 0.01) × 10^4^

### Protoplast regeneration

A protoplast suspension samples (0.1 mL) were plated on the agar medium A containing 0.4–1.2 M osmostabilizer (KCl, NH_4_Cl or sucrose) and incubated at 28 °C for maximum 12 days.

### Mutagenesis

Suspension of protoplasts (10 mL) obtained as described above was placed in a sterile Petri dish. Mutations were induced by exposure to ultraviolet (UV) irradiation (256 nm) at a 3-cm distance for 1–10 min. After UV irradiation, protoplast suspension samples (0.1 mL) were plated on the agar medium A supplemented with an osmostabilizer and ketoconazole (1–100 μM) and incubated at 28 °C for no more than 12 days.

### LCA bioconversion

For LCA bioconversion, medium B containing (g/L): soy flour—20, yeast extract—5, corn steep solids—10, K_2_HPO_4_—2, KH_2_PO_4_—16, MgSO_4_—0.5, FeSO_4_—0.02, TWEEN 80—0.1% (v/v) (pH 7.2) was inoculated with aliquot of the first generation mycelium (10%, v/v). LCA was added as a hot methanol solution [final solvent concentration did not exceed 2% (v/v)]. Bioconversion was performed aerobically on a rotary shaker (220 rpm) at 28 °C for a maximum of 168 h and steroid content was monitored daily by TLC and GC analyses as described below.

### Isolation of steroids

At the end of incubation period, steroids were extracted twice from the cultivation broth with equal volumes of ethyl acetate (EtOAc). The extracts were concentrated to 2–3 mL by rotary evaporation of the solvent under vacuum and the obtained crude residues were fractionated by means of silica gel column chromatography (Kollerov et al. 2008). The column (16 mm × 450 mm) with Silica gel 60 (0.040–0.063 mm) (Merck, Gernsheim, Germany) as a sorbent and chloroform/acetone/acetic acid mixtures of various percentage were applied. The individual compounds were analyzed by mass-spectrometry (MS), ^1^H and ^13^C nuclear magnetic resonance (NMR) methods as described below.

### Thin layer chromatography (TLC)

The samples of cultivation broth (1 mL) were extracted with 5 mL of EtOAc. The extracts were applied to the pre-coated TLC-sheets ALUGRAM SIL G/UV_254_ (Düren, Germany), developed in a mixture of CHCl_3_-acetone-CH_3_COOH (50:50:0.5, v/v). The staining of TLC plates was carried out by MnCl_2_ reagent followed by heating to 105 °C until color developed.

MnCl_2_ reagent: MnCl_2_ × 4H_2_O—0.2 g, H_2_O—30 mL, methanol—30 mL, H_2_SO_4_ (97%)—2 mL (dropwise).

### Gas chromatography (GC)

The samples (20 mL) of the cultivation broths were extracted with EtOAC (3 × 40 mL), the solvent was evaporated under vacuum and the residue was re-dissolved in EtOH (5 mL). BAs were analyzed in accordance with (Hayakawa et al. 1980). Trimethylsilyl derivatives of methyl esters of BAs were obtained as follows: 700 μL of 2 N solution of hydrogen chloride in methanol and 70 μL of 2,2-dimethoxypropane were added to the dry residue which was obtained after evaporation of the ethanol solution, the mixture was heated for 1 at 85 °C and evaporated to dryness under vacuum. Then, 100 µL of dehydrated methanol was added to the dried residue and again evaporated to dryness. Silanization was carried out by treatment with 600 μL of pyridine and bis (*N,O*-trimethylsilyl) trifluoracetamide (BSTFA) (2:1, v/v) for 40 min at 80 °C.

Analyses were performed on an HP 5890 chromatograph (Poway, USA), with SPB-1 quartz column (15 m × 0.25 mm), carrier gas (helium) flow—1.4 mL/min, column temperature—150–290 °C, and plasmic ionization detector with signal registration on HP 3396A integrator.

Retention times (R_t_): LCA—6.54 min; UDCA – 7.49 min.

### Mass-spectrometry (MS), ^1^H- and ^13^C-NMR spectroscopy

MS spectra were recorded on Bruker Esquire 3000 Plus spectrometer (Billerica, USA).

1H-NMR spectra were recorded on UNITY + 400 «Varian» (Palo Alto, USA) with working frequency 300 MHz on 1H NMR nucleus in DMSO-d6 and CDCl3. Tetramethylsilane was used as an internal standard.

Spectral data of UDCA metabolite formed by *G. zeae* strains:

3α,7β-Dihydroxy-5β-cholan-24-oic acid (ursodeoxycholic acid, UDCA): Mp 198 ^0^C [lit.[34] mp 196 ^0^C]; ^1^H-NMR (solvent d_6_-DMSO), selected signals, δ: 3.494 (2H, m, H-3β and H-7β); 0.980 (3H, s, CH_3_-19); 0.977 (3H, d; *J* = 6.2 Hz, CH_3_-21); 0.734 (3H, s, CH_3_-18) (Dangate et al. 2011). ^13^C-NMR (solvent d_6_-DMSO) δ: 176,882; 70.901; 70.721; 56.269; 55.335; 43.554; 43.292; 42.817; 40.340; 39.494; 37.379; 36.792; 35.432; 34.871; 33.947; 31.138; 30.798; 29.810; 28.358; 26.691; 22.690; 21.160; 17.687; 11.406. ESI–MS: 391.20 (calculated for [C_24_H_40_O_4_ + H]^+^: 391.56); [α]_D_ =  + 58 [lit. (Dangate et al. 2011) [α]_D_ =  + 59].

### Statistical analysis

Microsoft Excel was used for data processing. All the experiments were carried out in triplicate and each presented value was the average of three independent experiments. Standard deviations (SD) were estimated using the following equation and were shown as errors on the graphs and in the Table 1:

$${\text{SD}} = \sqrt {\frac{{\sum {\left| {x - \mu } \right|^{2} } }}{{\text{N}}}}$$

where ∑ means "sum of", x is a value in the data set, μ is the mean of the data set, and N is the number of data points in the population.

## Results

### Obtaining of *G. zeae* protoplasts

#### Effect of medium composition and mycelium age

The maximum yield of protoplasts (0.6 × 10^5^/mL) was obtained using a medium containing glucose and yeast extract (medium no.3), while glucose replacement with sucrose (medium no.1) resulted in smaller number of protoplasts generated from *G. zeae* mycelium (Table 1). Removal of yeast extract (medium no.2) negatively affected protoplast generation. The yield of protoplasts significantly decreased when the medium enriched with peptone (medium no. 4) or soy flour (medium no. 5) was used for the cultivation of fungus (Table 1).

**Fig. 2:**
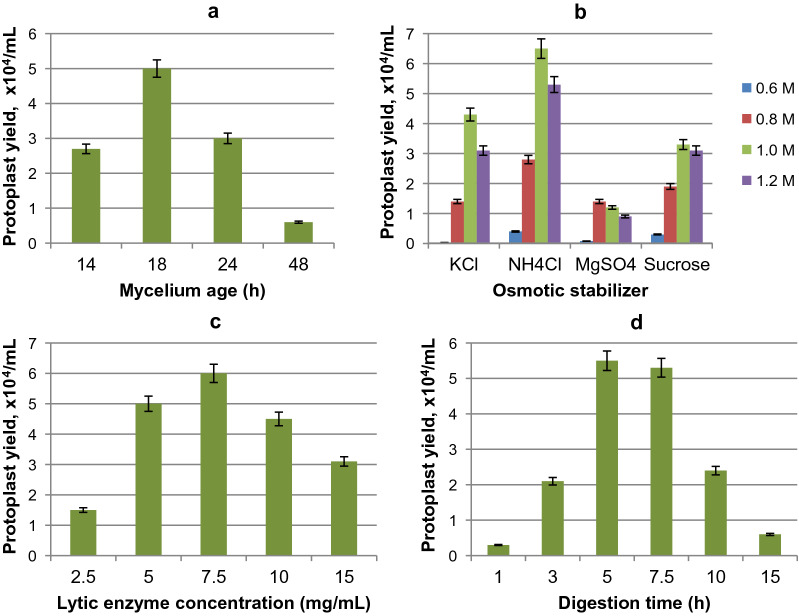
Influence of mycelium age (**a**), osmotic stabilizers (**b**), concentration of the lytic enzymes complex from *T. harzianum* (**c**) and digestion time (**d**) on *G. zeae* protoplast yield

**Fig. 3:**
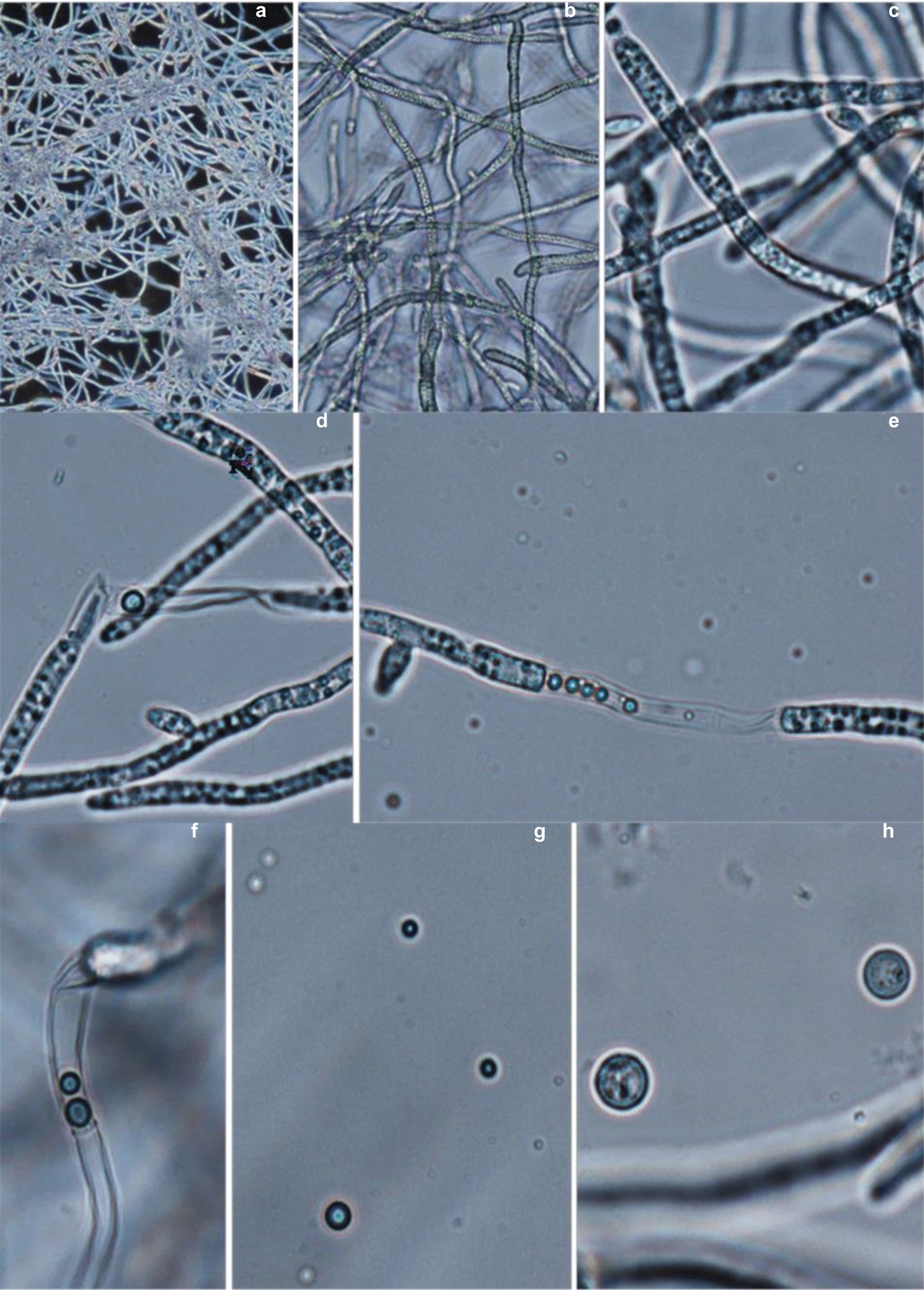
Phase-contrast micrographs of *Gibberella zeae* VKM F-2600 mycelium of second generation stage (18 h) grown in medium no. 3 (see Table 1) **(a**–**c)** and protoplasts released from 18 h fungal cells treated with *T. harzianum* lytic enzymes for 5 h (**d**–**h**): 100x (**a**), 400 (**b**) x, 1000x (**c-h**) (optical microscopy)

The highest yield of *G. zeae* protoplasts was observed when 18-h mycelium in the active growth phase was used for treatment with lytic enzymes (Figs. 2a, 3a–c), while younger (15-h) or older (24–48 h) mycelium was less suitable for protoplast formation (Fig. 2a).

#### Effect of osmotic stabilizers, lytic enzyme concentration and digestion time

In this study, we evaluated the effect of KCl, NH_4_Cl, MgSO_4_, and sucrose at various concentrations (0.6–1.2 M) on the integrity of *G. zeae* protoplasts. As shown in Fig. 2b, higher protoplast integrity was observed at a concentration of osmostabilizers of 1 M with the maximum level reached for ammonium chloride (Fig. 2b, 3h).

To obtain *G. zeae* protoplasts, we chose a cocktail of *T. harzianum* lytic enzymes, characterized by the activities of β-glucanase, cellulase, protease, and chitinase, and studied the effect of its various concentrations (2.5–15 mg/mL) and digestion time (1–15 h). Maximum yield of protoplasts was observed at the exposure of the lytic enzymes at concentration of 7.5 mg/mL for 5 h (Fig. 2c, d).

Thus, our results showed that the use of 18-h mycelium grown in a medium containing glucose and yeast extract, the treatment of cells with a cocktail of lytic enzymes at an optimal concentration of 7.5 mg/mL for 5 h, the use of 1 M ammonium chloride as an osmotic stabilizer provides up to 0.6 × 10^5^/mL of *G. zeae* protoplasts for further mutagenesis.

### Protoplast regeneration

**Fig. 4:**
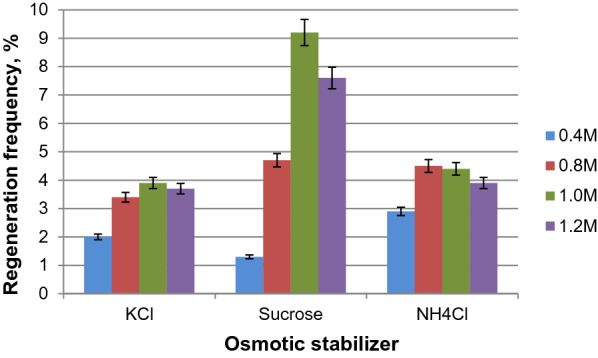
Influence of different osmotic stabilizers on regeneration frequency of *G. zeae* protoplasts

Regeneration rate of *G. zeae* protoplasts was largely determined by the choice of an osmotic stabilizer in the agar medium A. Ammonium chloride, which turned out to be the best osmotic stabilizer at the stage of protoplast release (Fig. 2b), nevertheless showed a smaller positive effect compared to sucrose at the regeneration stage (Fig. 4). The use of 1 M sucrose provided the highest regeneration frequency of *G. zeae* protoplasts (≥ 9%) (Fig. 4).

### Influence of ketoconazole and UV irradiation on protoplast viability

**Fig. 5:**
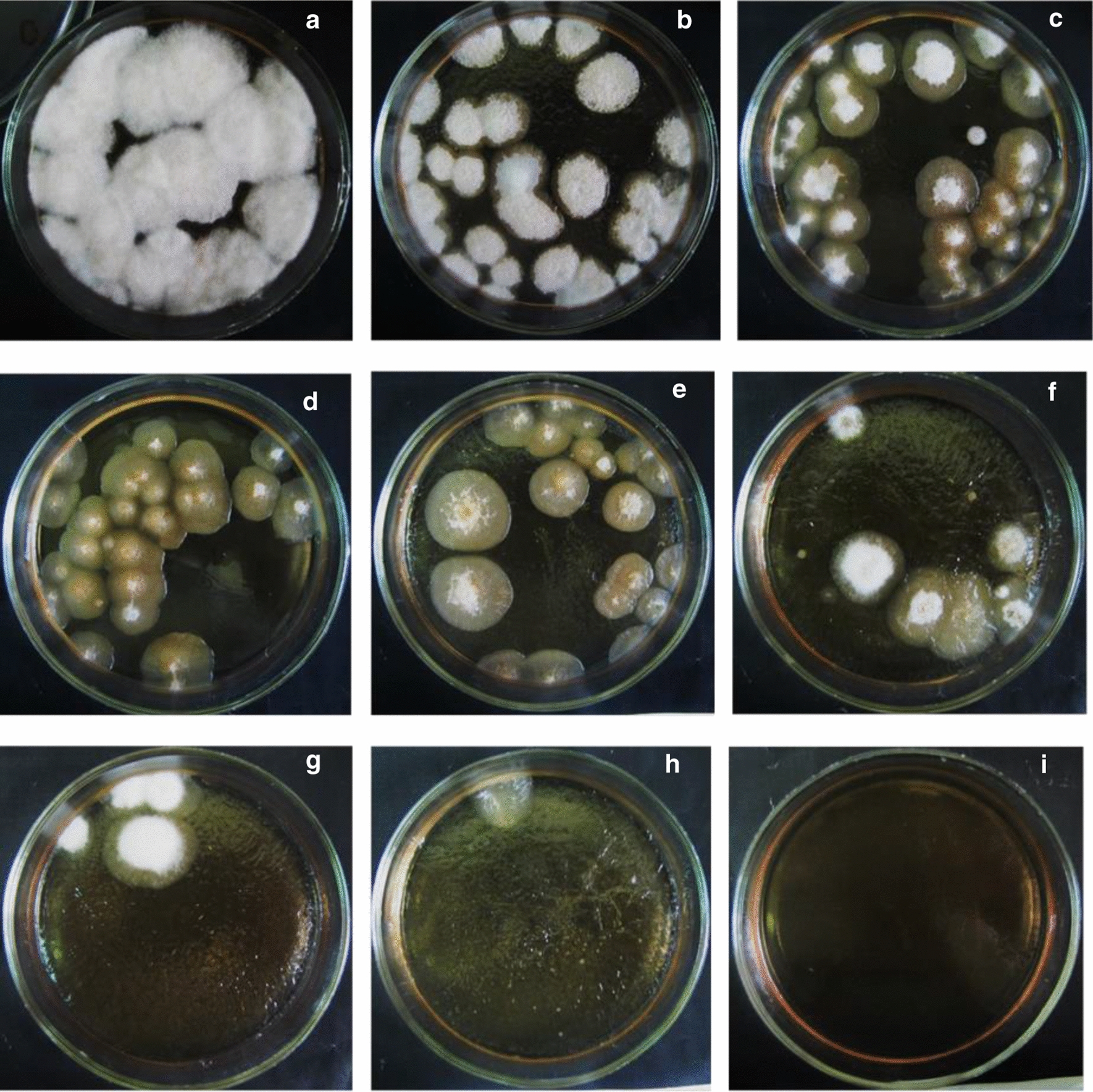
The regenerative morphology of the colonies of *G. zeae* protoplasts. 10 µL of protoplast suspension (0.5 × 10^5^/mL) was spread out on agar medium A supplemented with sucrose (1 M) and different concentrations of ketoconazole: 0 µM (**a**), 15 µM (**b**), 20 µM (**c**), 25 µM (**d**), 30 µM (**e**), 40 µM (**f**), 50 µM (**g**); 60 µM (**h**), 70 µM (**i**) and incubated at 28 °C for 12 days

**Fig. 6:**
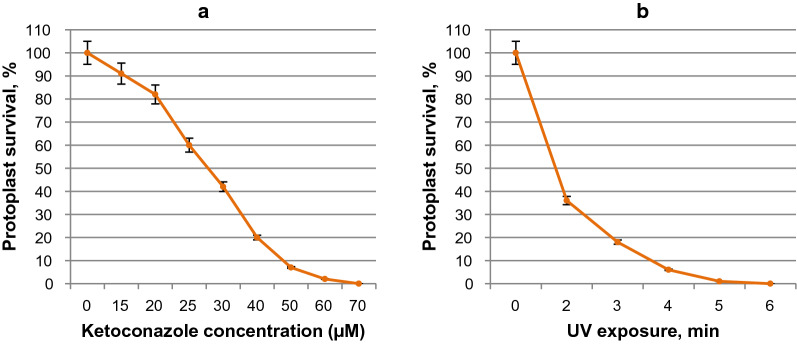
Effect of ketoconazole concentration (**a**) and UV exposure (**b**) on survival of *G. zeae* protoplasts

In this work, the influence of different concentration of ketoconazole (15–80 µM) on the viability of *G. zeae* protoplasts has been studied. Complete inhibition of the protoplast regeneration was observed at the fungicide concentration of 70 µM that was determined as the minimum inhibitory concentration (MIC) and used in further experiments on selection of ketoconazole-resistant mutant clones after UV mutagenesis and regeneration of fungal protoplasts (Figs. 5i, 6a).

**Fig. 7:**
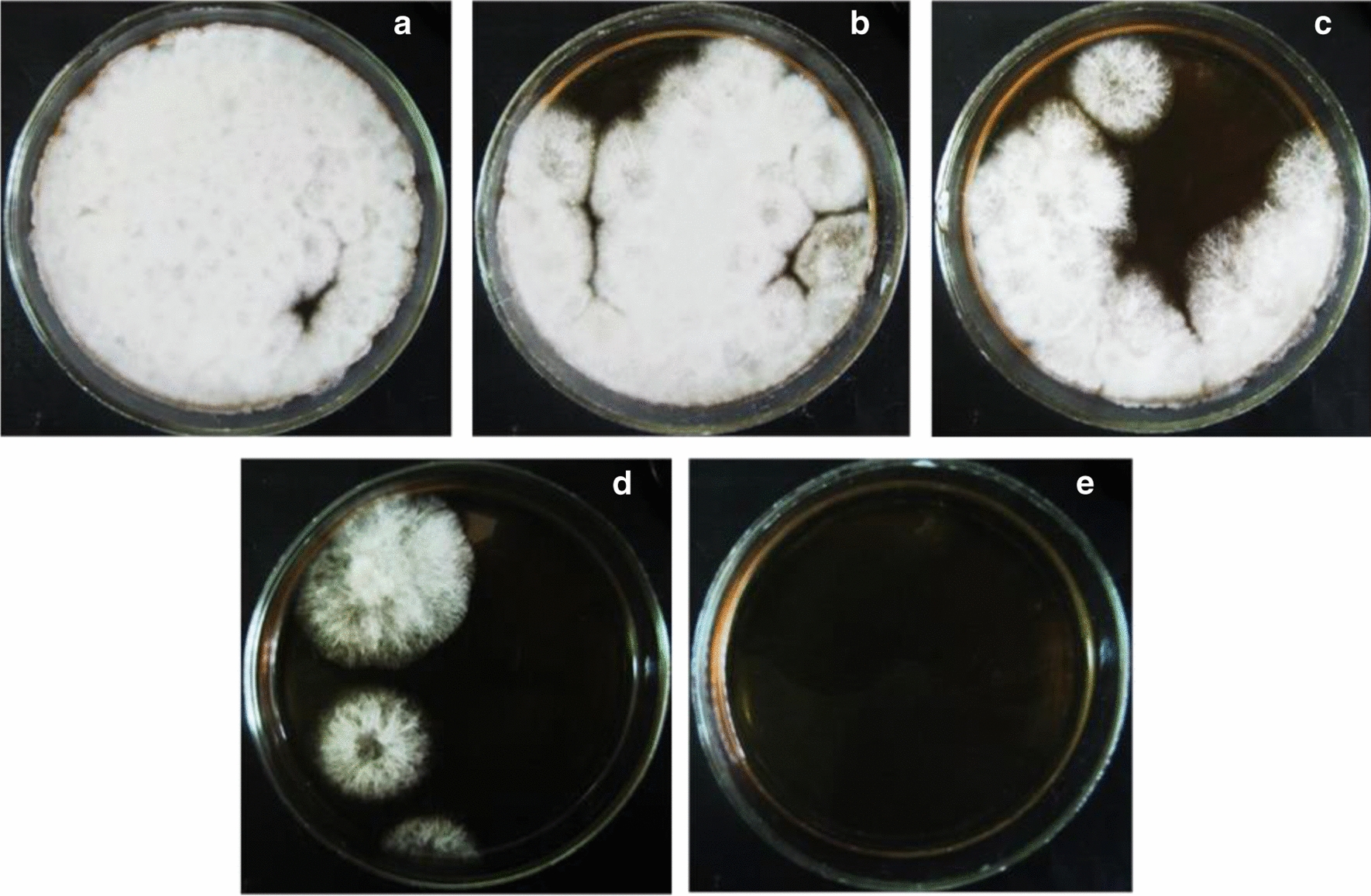
Regeneration of *G. zeae* protoplasts exposed to UV irradiation. 10 µL of protoplast suspension (0.5 × 10^5^/mL) was spread out on agar medium A with sucrose (1 M): without UV exposure (**a**) or exposed to UV for 2 min (**b**), 3 min (**c**), 5 min (**d**), 6 min (**e**) and incubated at 28 °C for 12 days

UV irradiation was used for the mutagenesis of *G. zeae* protoplasts. As follows from Fig. 6b and Fig. 7, an exposure of the mutagenic factor for 3.5–4 min provided survival of 6–12% fungal protoplasts that was reported to be optimum of viable cells in best numbers with possible only one or few mutations (Mukherjee and Sengupta 1986). Exposure to UV irradiation for more than 6 min resulted in complete loss of protoplast viability (Fig. 7e).

### Screening of mutant *G. zeae* strains activity towards LCA

**Fig. 8:**
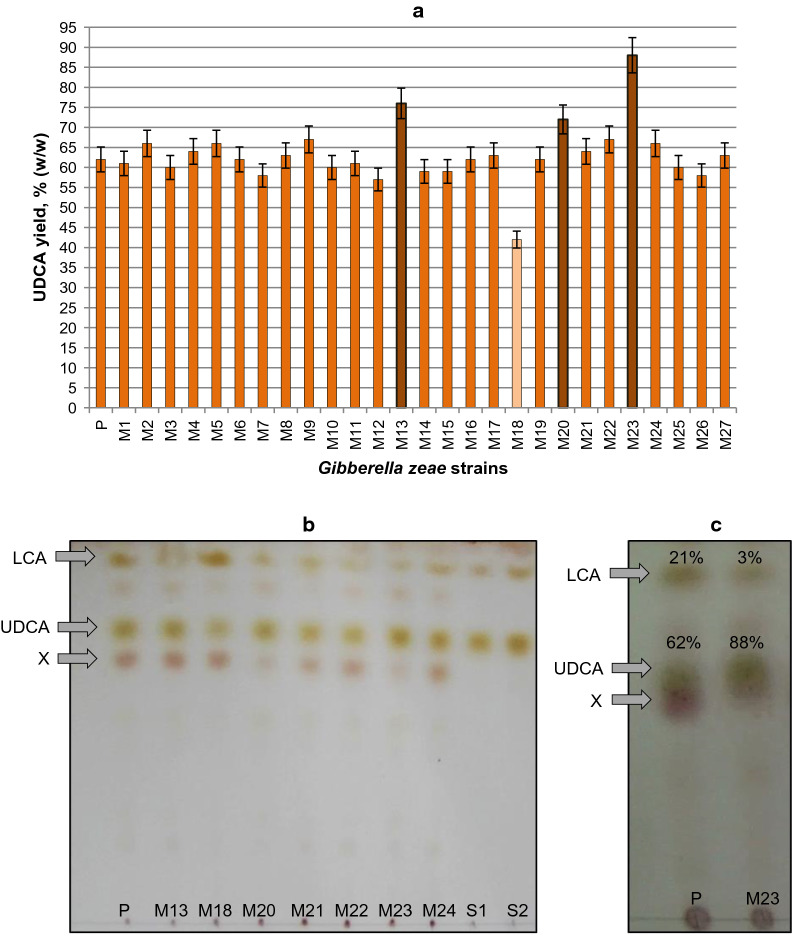
Comparative statistical analysis of LCA to UDCA conversion (144 h) by *G. zeae* parent (P) and mutant (M) strains (GC data of three independent experiments) (**a**). TLC profiles of 120 h (**b**) and 144 h (**c**) samples (~ 10 µg of steroids in the spot): S1, standard samples of LCA (0.5 mkg) and UDCA (7 mkg); S2, standard samples of LCA (1.0 mkg) and UDCA (8 mkg) (top to bottom); X–undefined metabolite (the structure of the product was not determined due to its low amount and concomitant formation of several metabolites). LCA initial concentration was 4 g/L

In total, 27 ketoconazole-resistant mutant clones have been isolated after protoplast mutagenesis and regeneration. The clones were screened for their biocatalytic activity towards LCA (4 g/L) and three mutant strains (M-13, M-20 and M-23) were revealed with superior activity (Fig. 8a).

According to TLC and GC analysis, the *G. zeae* M23 mutant strain exhibited the maximum 7β-hydroxylase activity towards LCA (4 g/L), providing a yield of the target UDCA 26% higher (up to 88% (w/w)) compared to the parent strain (Fig. 8). Noteworthy, the mutation influenced the selectivity of LCA to UDCA bioconversion: only about 3% (w/w) LCA retained unconverted in the case of mutant fungus, whilst it was about 21% (w/w) for the parent strain. Besides, the amount of the undefined metabolite X significantly decreased in the case of M23 mutant (Fig. 8c).

## Discussion

To improve the microbial synthesis of UDCA by ascomycete *G. zeae* VKM F-2600, various parameters involved in protoplast isolation and regeneration were studied, including medium composition, mycelial age, osmotic stabilizer, concentration of lytic enzymes and digestion time, concentration of the antifungal agent ketoconazole and UV irradiation dose.

The efficiency of the formation of *G. zeae* protoplasts was largely affected by the composition of the nutrient medium used for cultivating the mycelium of the second generation stage, which was higher when glucose and yeast extract were used as components of the medium. On the contrary, the application of peptone and soy flour led to a significant decrease in the yield of protoplasts (Table 1). This can be explained by possible alterations in the composition of the cell wall, which consists mainly of glucans, chitin, and glycoproteins and, as is well known, changes depending on the growth conditions (Wu and Chou 2019; Garcia-Rubio et al. 2020).

Mycelium age is regarded as one of the main factors influencing the release of protoplasts (Coelho et al. 2010; Wei et al. 2010). The structure of the fungal cell wall is highly dynamic, and may vary during the cell division, growth of fungi and hyphal branching (Li et al. 2017). In the case of *G. zeae*, the use of log growth phase mycelium ensured the maximum yield of protoplasts (Figs. 2a, 3a-c). This correlates with the literature data, which in most cases demonstrate better protoplast generation from actively growing mycelium (Harling et al. 1988). The age of mycelium was shown to be an important factor while protoplast obtaining from *F. graminearum* Fg99: mycelia in different times after culturing (6, 8, 12 and 14) were examined for protoplast preparation and the best result was obtained when the spores were left to germinate for 6 h (Moradi et al. 2013).

A sharp (more than fivefold) decrease in the yield of *G. zeae* protoplasts when using 48-h mycelium (Fig. 2a) may be associated with increased differentiation of the population, a change in the frequency of septation of hyphae, and the appearance of spore cells with a denser cell wall (Ren et al. 2018). As shown earlier, *C. lunatus* protoplasts obtained from actively growing mycelium were presumably more resistant to external influences compared to protoplasts obtained from cells of older fungi (Długoński et al. 1998).

The choice of an osmotic stabilizer can largely determine the efficiency of protoplast formation while maintaining their integrity (Rui and Morell 1993). Osmotic pressure stabilizers can balance the internal and external osmotic pressure of protoplasts that have lost cell wall protection and prevent the destruction of protoplasts (Harris 1982). When obtaining fungal protoplasts, inorganic salts (KCl, MgSO_4_, NaCl or NH_4_Cl) or sugars (sucrose, glucose or sorbitol) are often used as osmotic stabilizers (Zhou et al. 2008; Coelho et al. 2010).

The use of 1 M NH_4_Cl contributed to the best integrity of *G. zeae* protoplasts (Figs. 2b, 3h). The results differ from those reported for the fungal strains of *Pseudozyma flocculosa*, *Epulorhiza repens*, *Ceratorhiza* sp. and *Benjaminiella poitrasii,* in which higher protoplast integrity was achieved using 0.6 M KCl as an osmoprotector (Coelho et al. 2010; Cheng and Bélanger 2000; Chitnis and Deshpande 2002). The properties and composition of cell walls differ in different fungal species and may vary depending on environmental conditions, which, in turn, necessitates experimental selection of the osmotic stabilizers used and their concentrations, as well as lytic agents and lysis conditions for protoplast generation (Patil and Jadhav 2015; Gow et al. 2017; Li et al. 2017; Peng et al. 2016). Previously when protoplast obtaining from *G. zeae* 5373 and ATCC 20,273 strains, the most effective combinations of lytic enzymes were chitinase plus β-glucuronidase yielding up to 1 × 10^5^/mL protoplasts (Adams et al. 1987; Adams and Hart 1989).

It is known that when obtaining fungal protoplasts, the formation of spheroplasts (cells with partial cell wall) may be observed and it often depends on the choice of lytic enzymes and duration time (Li et al. 2017; Wang et al. 1988). In turn, the presence of spheroplasts can increase the percentage of their regeneration (Wagner and Wilkinson 1993; Cabo et al. 1986). Our goal was to obtain true intact protoplasts of *G. zeae* for their further mutagenesis, and for this purpose a lysing enzymes complex from *T. harzianum* contains β-glucanase, cellulase, protease, and chitinase activities necessary for complete removal of cell wall was chosen. This, probably, contributed to the production of mainly protoplasts than spheroplasts in the case of *G. zeae* that, in turn, could affect a relatively low degree of their regeneration frequency (1.5–9%) (Fig. 5).

The concentration of the *T. harzianum* lytic enzyme cocktail chosen in this work and the digestion time strongly influenced the release of *G. zeae* protoplasts. Application of 7.5 mg/mL for 5 h (Figs. 2c, d) ensured best result, whilst the exposure for more than 10 h led to 2.5–10 times lower protoplast release (Fig. 2d). Probably, the protease, which is a part of the digestive complex, contributed to destruction of *G. zeae* protoplasts causing extensive damage of their membrane proteins. As reported earlier, the protoplasts of various fungi were destroyed by proteinases upon the contact for more than 4 h (Kitamoto et al. 1988). Prolonged incubation with digestive mixtures resulted in lysis of the protoplasts of *E. repens*, *Ceratorhiza* sp. and *Geomyces* sp. (Coelho et al. 2010; Ren et al. 2018).

Protoplast regeneration is a cell-wall reconstruction and return to mycelial growth. Usually, the protoplasts of filamentous fungi are characterized by a low frequency of regeneration, ranging from 0.1 to 50.0% for different fungi, depending mainly on the fungal species and the composition of the regeneration medium (Li et al. 2017; Balasubramanian et al. 2003). Tests for fungal growth at different concentrations of osmotic stabilizers are needed because, in some cases, changing the concentration of ingredients in the regeneration media can significantly increase the frequency of protoplast regeneration (Coelho et al. 2010).

The highest regeneration frequency of *G. zeae* protoplasts (≥ 9%) was observed while using 1 M sucrose in the agar medium (Fig. 4). The positive effect of sucrose on protoplast regeneration was previously shown for *E. repens*, *Ceratorhiza* sp. and *Moniliophthora perniciosa* (previously *Crinipellis perniciosa*) fungal strains: the use of 0.5 M sucrose provided the best regeneration rate ranging from 5 to 10% (Coelho et al. 2010; Lima et al. 2003). 0.8 M sucrose was successfully used for regeneration of *G. zeae* 5373 and ATCC 20,273 protoplasts however if 0.8 M mannitol was used to suspend the protoplasts instead of 0.8 M sucrose, then the number of regenerated protoplasts decreased by approximately 20% (Adams et al. 1987; Adams and Hart 1989). For *P. flocculosa* the regeneration frequency of protoplasts reached 75% when 0.8 M sucrose was used as an osmotic stabilizer in agar medium (Cheng and Bélanger 2000). The protoplast yields of fungus *Antrodia cinnamomea* in the reaction mix with MgSO_4_ and KCl as osmotic stabilizers gradually decreased after 4-h digestion; on the other hand, sucrose (0.8 M) maintained a stable protoplast yield throughout the 9-h digestion (Garcia-Rubio et al. 2020).

Fungal protoplast mutation is known to be a fast and convenient method providing high sensitivity to stimulation, high mutation rate, and simplicity of the screening procedure (Peng et al. 2016). The use of *N*-methyl-*N'*-nitro-*N*-nitrosoguanidine (NTG) or UV light irradiation are the most common methods of fungal protoplast mutagenesis (Wilmańska et al. 1992; Shafique et al. 2009; Besoain et al. 2007). Successful use of UV irradiation was previously observed when obtaining various *G. zeae* 'Dewar', 65-338B, 251-15 and ATCC 20273 mutants including adenine-, arginine-, and histidine auxotrophs; a non-nutritional, heat-sensitive mutant; and an NADPH-dependent, glutamate dehydrogenase-deficient mutant (Leslie 1983).

In the present study, the optimum of UV exposure (3.5 min) as well as the MIC of ketoconazole (70 µM) hindering *G. zeae* protoplast regeneration have been defined (Figs. 5i, 6a). The imidazole antifungal agent is well known as an effective inhibitor of cytochrome P450 enzymes and previously it was used as a selective marker while screening of mutant strains of *C. lunatus* with increased steroid hydroxylase activity (Oh et al. 1993; Lu et al. 2007; Kollerov et al. 2010).

By combining the developed original procedure of *G. zeae* protoplast obtaining, UV mutagenesis and regeneration, 27 ketoconazole-resistant mutant clones were obtained. Comparative statistical analysis of LCA to UDCA conversion by *G. zeae* parent and mutant strains based on GC data of three independent experiments revealed three mutant strains (M-13, M-20 and M-23) capable of producing 10–30% more UDCA. One of the strains (M18) showed weaker than parent strain activity towards LCA (Fig. 8a). Other selected mutant strains did not differ from the parent fungus on their activity towards LCA (Fig. 8a). Among the tested strains, the mutant *G. zeae* M23 was chosen due to its superior target activity. The strain selectively converted LCA even at 4 g/L to give up to 88% of UDCA (Fig. 8c).

Up to date, the use of fungal protoplasts in the producing mutant strains with increased steroid hydroxylase activity was reported only for *C. lunatus* strains that are known mainly for their 11β-hydroxylase activity and applied in the synthesis of hydrocortisone. Using this approach, a mutant *C. lunata* KA91 had been obtained that demonstrated 42% higher production of 11β-hydroxylated products compared to parent strain (Lu et al. 2007). The selectivity of cortexolone to hydrocortisone conversion was increased when using mutant clones of *C. lunata* IM2901 (Wilmańska et al. 1992). In comparison with parent strain, *C. lunata* M4 mutant strain showed 25–30% higher yield of 11β-hydroxylated derivatives from 17α,21-diacetate cortexolone (Kollerov et al. 2010).

To our knowledge, no data on the improvement of steroid 7β-hydroxylase activity of the fungal strains via protoplast obtaining and mutagenesis have been reported so far. It should be noted that the absolute value of UDCA titer (3.52 g/L) reached using *G. zeae* M23 mutant strain obtained in this work significantly exceeds LCA to UDCA conversion rates ever reported for fungal strains, including *Fusarium equiseti* M-41 (a maximum UDCA yield of 0.35 g/L) (Sawada et al. 1982, 1986), *Penicillium* sp. TTUR 422 (FERM BP-5410) (maximum 0.25 g/L of UDCA) (Okamura and Matsui 1999) and the representatives of *Bipolaris*, *Cunninghamella*, *Cochliobolus* and *Gibberella* genera where UDCA yield did not exceed 0.9 g/L (Kollerov et al. 2013).

The results evidence that the method based on protoplast generation, UV mutagenesis and selection of ketoconazole-resistant mutant clones allows improvement of steroid hydroxylase activity of filamentous fungi and open up the prospects for effective microbial manufacturing of the value-added UDCA.

With high probability, we expect that 7β-hydroxylase from *G. zeae* relates to CYP monooxygenases. Despite the publications on the complete genome sequences of related strains of *G. zeae* and *F. graminearum* (Jurgenson et al. 2002; King et al. 2015), the 7β-hydroxylase gene has not yet been identified. To fill this gap, our next work will focus on the identification of the gene encoding steroid 7β-hydroxylase in *G. zeae* VKM F-2600 using transcriptomic analysis, cloning and heterologous expression, followed by a comparative analysis of the parental and mutant strain M23 for the presence of mutations in the sequences of the selected gene that may contribute to its overexpression.

## Data Availability

The data generated or analyzed during this study are included in this article.
